# A Flexible Coding Scheme Based on Block Krylov Subspace Approximation for Light Field Displays with Stacked Multiplicative Layers

**DOI:** 10.3390/s21134574

**Published:** 2021-07-04

**Authors:** Joshitha Ravishankar, Mansi Sharma, Pradeep Gopalakrishnan

**Affiliations:** Department of Electrical Engineering, Indian Institute of Technology Madras, Chennai 600036, India; ee19d401@smail.iitm.ac.in (J.R.); pradeepgopalakrishnan32@gmail.com (P.G.)

**Keywords:** light field, lossy compression, layered tensor 3D displays, convolutional neural network, Krylov subspace, low-rank approximation, randomized block Krylov singular value decomposition, rank analysis, rate distortion

## Abstract

To create a realistic 3D perception on glasses-free displays, it is critical to support continuous motion parallax, greater depths of field, and wider fields of view. A new type of *Layered* or *Tensor* light field 3D display has attracted greater attention these days. Using only a few light-attenuating pixelized layers (e.g., LCD panels), it supports many views from different viewing directions that can be displayed simultaneously with a high resolution. This paper presents a novel flexible scheme for efficient layer-based representation and lossy compression of light fields on layered displays. The proposed scheme learns stacked multiplicative layers optimized using a convolutional neural network (CNN). The intrinsic redundancy in light field data is efficiently removed by analyzing the hidden low-rank structure of multiplicative layers on a Krylov subspace. Factorization derived from Block Krylov singular value decomposition (BK-SVD) exploits the spatial correlation in layer patterns for multiplicative layers with varying low ranks. Further, encoding with HEVC eliminates inter-frame and intra-frame redundancies in the low-rank approximated representation of layers and improves the compression efficiency. The scheme is flexible to realize multiple bitrates at the decoder by adjusting the ranks of BK-SVD representation and HEVC quantization. Thus, it would complement the generality and flexibility of a data-driven CNN-based method for coding with multiple bitrates within a single training framework for practical display applications. Extensive experiments demonstrate that the proposed coding scheme achieves substantial bitrate savings compared with pseudo-sequence-based light field compression approaches and state-of-the-art JPEG and HEVC coders.

## 1. Introduction

Realistic presentation of a three-dimensional world on displays has been a long-standing challenge for researchers in the areas of plenoptics, light field, and full parallax imaging [1,2,3]. Glasses-free or naked-eye autostereoscopic displays have replaced stereoscopic displays which offer motion parallax for different viewing directions [4]. However, current naked-eye displays fall far short of truly recreating continuous motion parallax, greater depth-of-field, and a wider field-of-view for visual reality [5,6,7,8].

Designs based on a single display panel attached with a parallax barrier or special lens (lenticular screen or integral photography lens) usually suffer from inherent resolution limitations. The resolution for each view decreases with an increase in multiple viewing directions. Thus, supporting a full parallax visualization of the 3D scene is impractical [4]. On the other hand, both monitor-style and large-scale systems based on several projectors introduce a wide viewing approach, but such light field displays do not maintain a thin form factor and require ample space for the entire setup. Besides, such large-scale systems require costly hardware and a computationally expensive light field processing pipeline to reproduce high-quality views [9,10].

**Figure 1:**
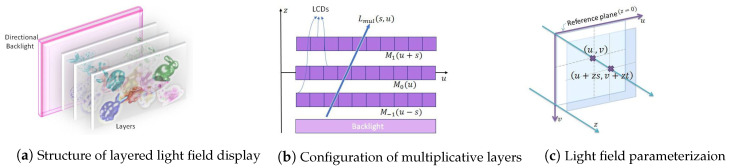
Light field is defined in 4-D space. The structure (**a**) and configuration (**b**) of layered light field display for constructing multiplicative layers are shown in 2-D for simplicity; (**c**) the light ray is parameterized by point of intersection with the (u,v) plane and the (s,t) plane located at a depth *z*.

Multi-layered or tensor light field displays offer an optimized solution to support direction-dependent outputs simultaneously, without sacrificing the resolution in reproducing dense light fields. This is deemed as a critical characteristic of glasses-free multi-view displays [5,11,12,13,14,15,16,17]. A typical structure of a layered 3D display is demonstrated in Figure 1a. It is composed of a few light-attenuating pixelized layers stacked with small intervals in front of a backlight. The transmittance of pixels on each layer can be controlled independently by carrying out light-ray operations (multiplication and addition) on the layered patterns. A multi-layered display with liquid crystal display (LCD) panels and a backlight is implemented by performing multiplicative operations on layers, and additive layers are fabricated with holographic optical elements (HOEs) and projectors [14]. With this structure, the layer patterns allow light rays to pass through different combinations of pixels depending on the viewing directions. As shown in Figure 1b, multiplicative layer patterns overlap with different shifts in observed directions. With layered displays, we can precisely reproduce multi-view images or an entire light field simultaneously with high resolution by considering just a few light attenuating layers. Further, compactly representing the light field using transmittance patterns of only a few layers offers display adaptation. Directed from a target light field, transmittance patterns of stack pixelized layers are useful in determining the multi-view images required for various display types design with light fields, such as projection displays [9,18], mixed reality head-mounted displays [19,20,21], table-top displays, and mobile platforms [22]. It is critical to analyze the intrinsic redundancy in light fields to generate an efficient 3D production and content delivery pipeline using multi-layer-based approaches. This is essential to efficiently represent (multiplicative or additive) stacked layers for the desired light-field output.

In this paper, we address the problem of light field dimensionality reduction for existing multi-layer or tensor 3D displays. The CNN is employed to optimize multiplicative layers obtained from light field data. CNN-based methods are proven to be better in terms of balance between computation time and accuracy than the previous analytical optimization approaches based on non-negative tensor factorization [5,14,17,18]. A novel coding scheme for the multiplicative layers is proposed based on a randomized block Krylov singular value decomposition framework. The proposed algebraic representation of stacked multiplicative layers on the Krylov subspace approximates the hidden low-rank structure of the light field data. Factorization derived from BK-SVD efficiently exploits the high spatial correlation between multiplicative layers and approximates the light field with varying low ranks. The encoding with HEVC further eliminates inter-layer and intra-layer redundancies in low-rank approximated representation of multiplicative layers and considerably improves the compression efficiency. By choosing varying ranks and quantization parameters, the scheme is flexible to optimize the bandwidth depending on the display device availability for a given target bitrate. This allows the delivery of 3D contents with the limited hardware resources of layered displays and best meets the viewers’ preferences for depth impression or visual comfort.

The majority of existing light field coding approaches are not directly applicable for multi-layered displays. Several coding approaches extract the SAIs of the light field and interpret them as a pseudo video sequence [23,24,25,26,27,28]. They utilize existing video encoders, like HEVC [29] or MV-HEVC, for inter and intra-frame hybrid prediction. View estimation based methods [30,31,32,33] reconstruct the entire light field from a small subset of encoded views and save bandwidth. However, such algorithms fail to remove redundancies among adjacent SAIs and restrict prediction to the local or frame units of the encoder. In addition, learning-based view-synthesis methods for light field compression [34,35,36,37,38,39] require large-scale and diverse training samples. To reconstruct high quality views, a significant fraction of the SAIs have to be used as references.

The algorithms that exploit low rank structure in light field data follow disparity based models [40,41]. Jiang et al. [40] proposed a HLRA method that aligns light field sub-aperture views using a set of homographies estimated by analyzing how disparity across views varies from different depth planes. The HLRA may not optimally reduce the low-rank approximation error for light fields with large baselines. A similar issue is noticeable in the parametric disparity estimation model proposed by Dib et al. [41]. This method requires a dedicated super-ray construction to deal with occlusions. Without proper alignment, it cannot precisely sustain the angular dimensionality reduction (based on low-rank approximation). Recently, geometry-based schemes have also gained popularity for efficient compression at low-bit rates [42,43,44]. Such schemes use light field structure/multi-view geometry and are not suitable for coding layer patterns directly for multi-layer 3D display applications.

Methods that explicitly consider the content of light field data for compression [45,46] also do not work in present settings. Liu et al. [45] compress plenoptic images by classifying light field content into three categories based on texture homogeneity and use corresponding Gaussian process regression-based prediction methods for each category. The performance depends on scene complexity, accurate classification into prediction units, and sophisticated treatment is required to handle the texture and edge regions of the lenslet image. Similarly, the GNN-based scheme presented by Hu et al. [46] separates high-frequency and low-frequency components in sub-aperture images which are then encoded differently. This scheme needs an accurate parameter estimation model and discards specific frequency components permanently. All these coding techniques are not explicitly designed for layered light field displays. They also usually train a system (or network) to support only specific bitrates during the compression.

Different from existing approaches, our proposed Block Krylov SVD based lossy compression scheme works for layered light-field displays with light-ray operations regulated using stacked multiplicative layers and a CNN-based method. The concept of “one network, multiple bitrates” motivates us to achieve the goal of covering a range of bitrates, leveraging the generality of low-rank models and data-driven CNNs for different types of display devices. The proposed coding model does not just support multi-view/light field displays; it can also complement existing light-field coding schemes, which employ different networks to encode light field images at different bit rates. Our experiments with real light field data exhibit very competitive results. The main contributions of the proposed scheme are:

- A novel scheme for efficient layer-based representation and lossy compression of light fields on multi-layered displays. The essential factor for efficient coding is redundancy in multiplicative layers patterns, which has been deeply analyzed in the proposed mathematical formulation. The source of intrinsic redundancy in light field data is analyzed on a Krylov subspace by approximating hidden low-rank structure of multiplicative layers considering different ranks in factorization derived from Block Krylov singular value decomposition (BK-SVD). The scheme efficiently exploits the spatial correlation in multiplicative layers with varying low ranks. Further, encoding with HEVC eliminates inter-frame and intra-frame redundancies in the approximated multiplicative layers and improves compression efficiency.
- The proposed scheme is flexible to realize a range of multiple bitrates within an integrated system trained using a single CNN by adjusting the ranks of BK-SVD representation and HEVC quantization parameters. This critical characteristic of the proposed scheme sets it apart from other existing light field coding methods, which train a system (or network) to support only specific bitrates during the compression. It can complement existing light-field coding schemes, which employ different networks to encode light field images at different bit rates.
- The proposed scheme could flexibly work with different light-ray operations (multiplication and addition) and analytical or data-driven CNN-based methods. It is adaptable for a variety of holographic and multi-view light field displays. This would enable deploying the concept of layered displays on a variety of computational or multi-view auto-stereoscopic platforms, head-mounted displays, table-top or mobile platforms by optimizing the bandwidth for a given display target bitrate and desired light-field output. Further, the study is important to understand the kinds of 3-D contents that can be displayed on a layered light-field display with limited hardware resources.
- The proposed low-rank coding scheme compresses the optimal multiplicative layered representation of the light field, rather than the entire light field views. Since the proposed scheme uses just three multiplicative layers, it is advantageous in terms of computation speed, bytes written to file during compression, bitrate savings, and PSNR/SSIM performance. We believe that this study triggers new research that will lead to a profound understanding in applying mathematically valid tensor-based factorization models with data-driven CNNs for practical compressive light field synthesis and formal analysis of layered displays.

The rest of this article is organized into three major sections. The proposed mathematically valid representation and coding scheme for multi-layered displays is described in Section 2. Section 3 describes our implementation, experiments, results, and evaluation. Finally, Section 4 concludes the article with a discussion on comprehensive findings of our proposed scheme and implication of future extension.

## 2. Proposed Coding Scheme for Multi-Layered Displays

**Figure 2:**
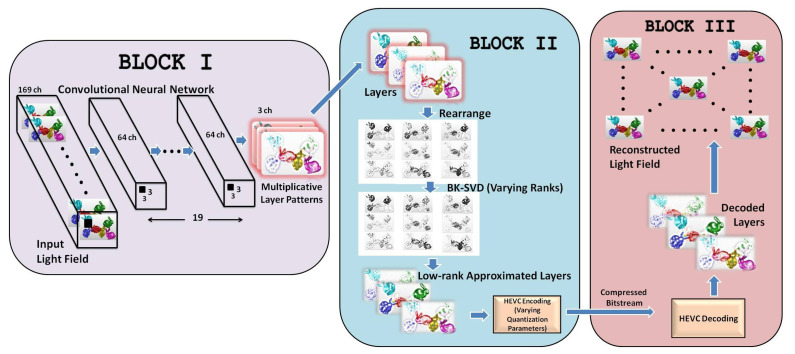
The complete workflow of the proposed scheme comprising of three prime components: conversion of light field views into multiplicative layers, low-rank approximation of layers and HEVC encoding, and the decoding of the approximated layers followed by the reconstruction of the light field.

The workflow of our proposed representation and coding scheme is illustrated in Figure 2. The proposed scheme is divided into three major components. The first component (*BLOCK I*) represents a convolutional neural network that converts the input light field views into three multiplicative layers. In the second component (*BLOCK II*), the intrinsic redundancy in light field data is efficiently removed by analyzing the hidden low-rank structure of multiplicative layers on a Krylov subspace by varying BK-SVD ranks. This followed by the encoding of low-rank approximated multiplicative layers using HEVC effectively eliminates inter-frame and intra-frame redundancies. In the last component (*BLOCK III*), the light field is reconstructed from the decoded layers, as depicted in Figure 2. Each component of the proposed representation and compression scheme is described in the following sections.

### 2.1. Light Field Views to Stacked Multiplicative Layers

In the first component, the proposed scheme generates optimized multiplicative layers from real light field data. The four-dimensional light field is parameterized by the coordinates of the intersections of light rays with two planes [47,48]. The coordinate system is denoted by (u,v) for the first plane and (s,t) for the second plane (Figure 1c). In this system, a light ray first intersects the uv plane at (u,v) and then the st plane at coordinate (s,t). The light field is represented by L(u,v,s,t). If the (s,t) plane is located at depth *z*, the ray will have coordinates (u+zs,v+zt).

Multiplicative layers [5] are light attenuating panels that are stacked in evenly spaced intervals in front of a backlight as shown in Figure 1. The transmittance of a layer $M_{z}$ is given by $M_{z}\left(u,v\right)$ and a light ray emitted from this display will have its intensity normalized by the intensity of the backlight and can be expressed as

(1) $$L_{mul}\left(s,t,u,v\right)=\prod_{z\in Z}M_{z}\left(u+zs,v+zt\right).$$

Here, we assume that a light-field display is composed of three layers located at Z={−1,0,1}, where z∈Z. It must be noted that *z* corresponds to the disparity among the directional views, rather than the physical length [14]. The multiplicative layers need to be optimized to display a 3-D scene. The optimization goal for the layer patterns is given by

(2) $$\underset{M_{z}|z\in Z}{\mathrm{argmin}}\sum_{s,t,u,v}{∥L\left(s,t,u,v\right)-L_{mul}\left(s,t,u,v\right)∥}^{2},$$

where L(s,t,u,v) depicts the light field emitted from the display.

We utilize a CNN to generate optimal multiplicative layers from the input light field images. The CNN-based optimization method proves to be better in terms of the balance between computation time and accuracy than the previous analytical optimization approach based on non-negative tensor factorization (NMF) methods [5,14,17,18]. Analytical methods obtain three multiplicative layers of an input light field by carrying out the optimization one layer at a time and repeating it for all layers until convergence [14]. The solutions are updated in an iterative manner, with a trade-off between computation time (number of iterations) and accuracy of the solution. On the other hand, CNN-based methods are proven to be faster and produce better accuracy, as well [14]. We have discussed the merits of using a convolutional neural network over analytical methods for layer pattern optimization in Section 3.5. Optimization for the three layer patterns from the full light field using CNN can be mapped as

(3) f:L→M,

where M represents a tensor containing all the pixels of $M_{z}\left(u,v\right)$ for all z∈Z.

Similarly, the mappings from the layers patterns to the light field $L_{mul}\left(s,t,u,v\right)$ can be rewritten as   

(4) $$f_{\mathrm{mul}}:M\to L_{\mathrm{mul}},$$

where $L_{\mathrm{mul}}$ represents all the light rays in $L_{mul}\left(s,t,u,v\right)$. During training, a CNN minimizes the squared error loss given by

(5) $$\underset{f}{\mathrm{argmin}}{∥L-L_{\mathrm{mul}}∥}^{2}.$$

**Figure 3:**
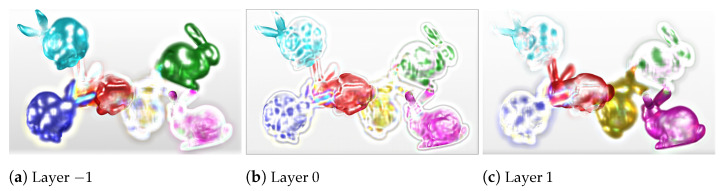
The three optimal multiplicative layers obtained from CNN (with 20 convolutional layers, trained for 20 epochs with a learning rate of 0.0001, and batch size 15) for *Bunnies* data (*BLOCK I* of the proposed scheme).

Note that $L_{\mathrm{mul}}=$$f_{mul}$(*f*(L)), where *f*(L) is performed by the CNN to produce the multiplicative layer patterns M, and $f_{mul}$ reconstructs light field $L_{\mathrm{mul}}$ from M. Tensors L and $L_{\mathrm{mul}}$ have 169 channels corresponding to the 13 × 13 views of the light field image, and tensor M has three channels corresponding to the three layer patterns of the multiplicative layered display. The final trained network can convert the input light field views into three optimized output layer patterns. This is the first component in our proposed coding scheme (*BLOCK I* in Figure 2). Figure 3 illustrates the CNN-produced multiplicative layers of the *Bunnies* light field [49].

### 2.2. Low Rank Representation and Coding of Stacked Multiplicative Layers on Krylov Subspace

The key idea of the proposed scheme is to remove the intrinsic redundancy in light field data by analyzing the hidden low-rank structure of multiplicative layers. The second component of our workflow (*BLOCK II* in Figure 2) involves this low-rank representation of optimized multiplicative layers. We represented the layers compactly on a Krylov subspace and approximated them using Block Krylov singular value decomposition (BK-SVD).

The Randomized Simultaneous Power Iteration method has been popularized to approximate the singular value decomposition (SVD) of matrices [50,51]. This simultaneous iteration approach can optimally achieve the low-rank approximation of a matrix within (1+ϵ) of optimal for spectral norm error by quickly converging in $\tilde{O}\left(\frac{1}{\epsilon }\right)$ iterations for any matrix. However, it may not guarantee a strong low-rank approximation or return high-quality principal components of the matrix. To improve this, Cameron Musco and Christopher Musco introduced a simple randomized block Krylov method [52], which can further improve the accuracy and runtime performance of simultaneous iteration. Experimentally, the randomized block Krylov SVD (BK-SVD) performs fairly better in just $\tilde{O}\left(\frac{1}{\sqrt{\epsilon }}\right)$ iterations. The BK-SVD approach is closely related to the classic Block Lanczos algorithm [53,54] and outputs a nearly optimal approximation for any matrix, independent of singular value gaps.

In our proposed scheme, we denote each multiplicative layer pattern produced by the CNN as $M_{z}\in R^{m\times n\times 3}$, where z∈{−1,0,1}. The red, green, and blue color channels of the layer *z* are denoted as $M_{z}^{r}$, $M_{z}^{g}$, and $M_{z}^{b}$, respectively. We constructed the matrices $B^{ch}\in R^{3m\times n}$, where ch∈{r,g,b}, as

(6) $$B^{r}=\left(\begin{array}{c} \;\;M_{-1}^{r}\\ M_{0}^{r}\\ M_{1}^{r} \end{array}\right)\;B^{g}=\left(\begin{array}{c} \;\;M_{-1}^{g}\\ M_{0}^{g}\\ M_{1}^{g} \end{array}\right)\;B^{b}=\left(\begin{array}{c} \;\;M_{-1}^{b}\\ M_{0}^{b}\\ M_{1}^{b} \end{array}\right).$$

**Figure 4:**
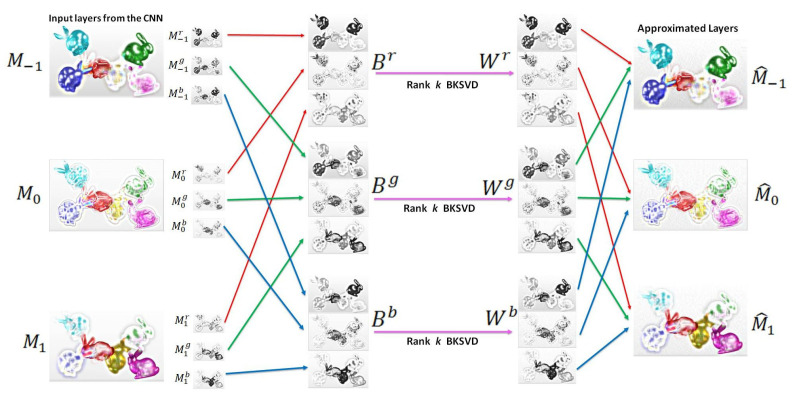
The BK-SVD procedure adopted in the proposed scheme. The layers $M_{z}$, z∈{−1,0,1} are split into color channels and rearranged to form matrices $B^{ch}$, ch∈{r,g,b}. The rank *k* approximation of $B^{ch}$ results in $W^{ch}$, which are then divided and rearranged to attain the approximated layers.

The intrinsic redundancies in multiplicative layers of the light field can be effectively removed by following low-rank BK-SVD approximation in a Krylov subspace for each $B^{ch}$, ch∈{r,g,b} (Figure 4). For simplicity, we will denote $B^{ch}$ as *B* in the following mathematical formulation.

Given a matrix $B\in R^{c\times d}$ of rank *r*, the SVD can be performed as $B=U\Sigma V^{T}$, where the left and right singular vectors of *B* are the orthonormal columns of $U\in R^{c\times r}$ and $V\in R^{r\times d}$, respectively. $\Sigma \in R^{r\times r}$ is a positive diagonal matrix containing $\sigma_{1}\geq \cdot \cdot \cdot \geq \sigma_{r}$, the singular values of *B*. Conventional SVD methods are computationally expensive. Thus, there is substantial research done on randomized techniques to achieve optimal low-rank approximation [50,51,52]. The recent focus has shifted towards methods that inherently do not depend on the properties of the matrix or the gaps in its singular values. In our present formulation of low-rank light field layer approximation, we seek to find a subspace that closely captures the variance of *B*’s top singular vectors and avoids the gap dependence in singular values. We target spectral norm low-rank approximation error of *B*, which is intuitively stronger. It is defined as

(7) $${∥B-WW^{T}B∥}_{2}\leq \left(1+\epsilon \right){∥B-B_{k}∥}_{2},$$

where *W* is a rank *k* matrix with orthonormal columns $w_{1},\cdot \cdot \cdot ,w_{k}$. In a rank *k* approximation, only the top *k* singular vectors of *B* are considered relevant and the spectral norm guarantee ensures that $WW^{T}B$ recovers *B* up to a threshold ϵ.

Traditional Simultaneous Power Iteration algorithms for SVD initialized with random start vectors achieve the spectral norm error (7) in nearly $\tilde{O}\left(\frac{1}{\epsilon }\right)$ iterations. Block Krylov SVD approach presented in Reference [52], a randomized variant of the Block Lanczos algorithm [53,54], guarantees to achieve the same in just $\tilde{O}\left(\frac{1}{\sqrt{\epsilon }}\right)$ iterations. This not only improves runtime for achieving spectral norm error (7), but guarantees substantially better performance in practical situations, where gap independent bound is critical for excellent accuracy and convergence. In the present formulation, we start with a random matrix $\Pi ∼N{\left(0,1\right)}^{d\times k}$ and perform Block Krylov Iteration by working with the Krylov subspace,

(8) $$K=\left[\begin{array}{c} \Pi \;B_{z}\Pi \;B_{z}^{2}\Pi \;B_{z}^{3}\Pi \cdot \cdot \cdot B_{z}^{q}\Pi \end{array}\right].$$

We take advantage of low degree polynomials that allow us to compute fewer powers of *B* and improve performance with fewer iterations. We construct $p_{q}\left(B\right)\Pi$ for any polynomial $p_{q}\left(\cdot \right)$ of degree *q* by working with Krylov subspace *K*. The approximation of matrix *B* done by projecting it onto the span of $p_{q}\left(B\right)\Pi$ is similar to the best *k* rank approximation of *B* lying in the span of the Krylov space *K*. Thus, the nearly optimal low-rank approximation of *B* can be achieved by finding this best rank *k* approximation. Further, to ensure that the best spectral norm error low-rank approximation lies in a specific subspace (i.e., in the span of *K*), we orthonormalize the columns of *K* to obtain $Q\in R^{c\times qk}$ using the QR decomposition method [55]. We take SVD of matrix $S=Q^{T}BB^{T}Q$ where $S\in R^{qk\times qk}$, for faster computation and accuracy. The rank *k* approximation of *B* is matrix *W*, which is obtained as

$$W=Q{\bar{U}}_{k},$$

where ${\bar{U}}_{k}$ is set to be the top *k* singular vectors of *S*. Consequently, the rank *k* block Krylov approximation of matrices $B^{r}$, $B^{g}$, and $B^{b}$ are $W^{r}$, $W^{g}$, and $W^{g}$, respectively. The $W^{ch}\in R^{x\times y}$ for every color channel ch. The approximated layers ${\hat{M}}_{z}\;,\;z\in \left\{-1,0,1\right\}$ are obtained by drawing out the color channels from the approximated $W^{ch}$ matrices.

We sectioned out the rows uniformly as

(9a) $$\begin{array}{c} {\hat{M}}_{-1}^{ch}=W^{ch}\left[1:x\;\;,\;\;1:y\;\;\right], \end{array}$$

(9b) $$\begin{array}{c} {\hat{M}}_{0}^{ch}=W^{ch}\left[x+1:2x\;\;,\;\;1:y\;\;\right], \end{array}$$

(9c) $$\begin{array}{c} {\hat{M}}_{1}^{ch}=W^{ch}\left[2x+1:3x\;\;,\;\;1:y\;\;\right]. \end{array}$$

The red, green, and, blue color channels are then combined to form each approximated layer, ${\hat{M}}_{-1}$, ${\hat{M}}_{0}$, and ${\hat{M}}_{1}$. These three block Krylov approximated layers are subsequently encoded using HEVC.

### 2.3. The Encoding of Rank-Approximated Layers

The High Efficiency Video Coding (HEVC) [29] is the latest international standard for video compression. It was standardized by ITU-T Video Coding Experts Group and the ISO/IEC Moving Picture Experts Group. It achieves improved compression performance over its predecessors, with at least a 50% bit-rate reduction for the same perceptual quality.

The encoding algorithm of HEVC flexibly divides each frame of the video sequence into square or rectangular blocks. This block partitioning information is relayed to the decoder. The first video frame is coded using only intra prediction (spatial prediction from other regions of the same frame), and succeeding frames are predicted from one, two, or more reference frames using inter and intra prediction. Motion-compensated prediction is used to remove the temporal redundancy during inter prediction.

**Figure 5:**
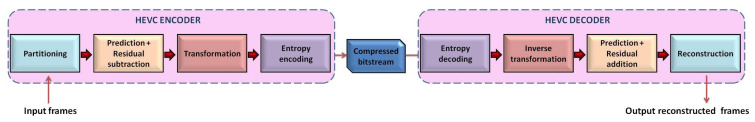
The workflow of the encoding and decoding steps of HEVC codec.

In our proposed scheme, we converted the block Krylov approximated layers into the YUV420 format. We then used HEVC encoder for various QPs to remove inter and intra redundancies of the low-rank approximated layers and compress them into a bitstream. A linear spatial transform is applied to the residual signal information between the intra or inter predictions. The transform coefficients are then scaled and quantized. The HEVC performs quantization of the coefficients using QP values in the range of 0–51. To make the mapping of QP values to the step sizes approximately logarithmic, the quantizer doubles step size each time the QP value increases by 6. The scaled and quantized transform coefficients are entropy coded and transmitted to the decoder along with the prediction information. The workflow of the entire encoding process is depicted in Figure 5.

### 2.4. The Decoding and Reconstruction of the Light Field

This section reports the decoding procedure of compressed layers and the reconstruction of light field (*BLOCK III* in Figure 2). The HEVC decoder performs entropy decoding of the received bitstream. It then reconstructs the quantized transform coefficients by inverse scaling and inversely transforming the residual signal approximation. The residual signal is added to the prediction, and this result is fed to filters to smooth out the artifacts introduced because of the block-wise processing and quantization. Finally, the predicted frames are stored in the decoder buffer to predict successive frames.

We decoded three layers from the bitstream and converted them into the RGB format. The decoded layers are denoted as ${\overset{‵}{M}}_{z}$, where z∈{−1,0,1} depicts the depth of the layers.

The SAIs are reconstructed from these decoded layers in the following way. Let $I_{\left(s^{*},t^{*}\right)}\in R^{m\times n}$ be the sub-aperture image at the viewpoint $\left(s^{*},t^{*}\right)$ and the intensity at pixel location (u,v) be $I_{\left(s^{*},t^{*}\right)}\left(u,v\right)$, where u∈[1,m] and v∈[1,n], u,v∈Z. Since we aim to reconstruct the inner 13 × 13 views of the light field, $s^{*},t^{*}$ are integers such that $-6\leq s^{*},t^{*}\leq 6$. This SAI $I_{\left(s^{*},t^{*}\right)}$ is obtained by translating the decoded layers to ${\overset{‶}{M}}_{z}$ and performing an element-wise product of the color channels of the translated layers. For a particular view $\left(s^{*},t^{*}\right)$, the translation of every *z*th layer ${\overset{‵}{M}}_{z}$, to ${\overset{‶}{M}}_{z}$ is carried out as

(10) $${\overset{‶}{M}}_{z(s^{*},t^{*})}\left(u,v\right)={\overset{‵}{M}}_{z}\left(u+zs^{*},v+zt^{*}\right).$$

Thus, the three translated layers for every viewpoint $\left(s^{*},t^{*}\right)$ are

$$\begin{array}{cc} & {\overset{‶}{M}}_{-1(s^{*},t^{*})}\left(u,v\right)={\overset{‵}{M}}_{-1}\left(u-s^{*},v-t^{*}\right),\\ & {\overset{‶}{M}}_{0(s^{*},t^{*})}\left(u,v\right)\;\;\;={\overset{‵}{M}}_{0}\;\left(u,v\right),\\ & {\overset{‶}{M}}_{1(s^{*},t^{*})}\left(u,v\right)\;\;\;={\overset{‵}{M}}_{1}\left(u+s^{*},v+t^{*}\right). \end{array}$$

An element-wise product of each color channel ch∈{r,g,b}, of the translated layers gives the corresponding color channel of the sub-aperture image.

(11) $$I_{\left(s^{*},t^{*}\right)}^{ch}={\overset{‶}{M}}_{-1(s^{*},t^{*})}^{ch}⊙{\overset{‶}{M}}_{0(s^{*},t^{*})}^{ch}⊙{\overset{‶}{M}}_{1(s^{*},t^{*})}^{ch}.$$

**Figure 6:**
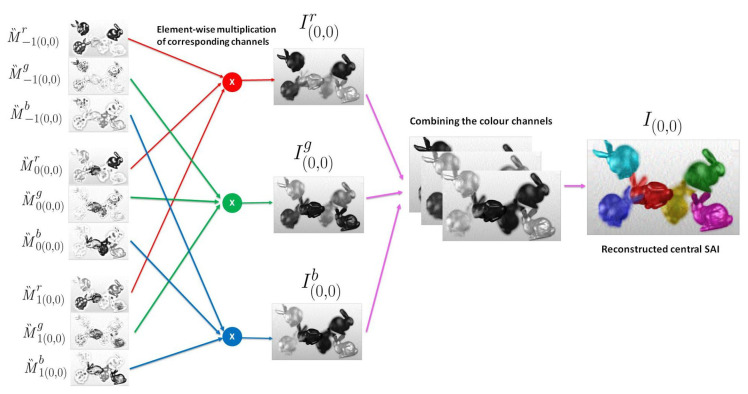
For the central viewpoint $\left(s^{*},t^{*}\right)=\left(0,0\right)$, the decoded layers ${\overset{‵}{M}}_{z}$ are translated to ${\overset{‶}{M}}_{z(0,0)}$. The corresponding color channels are multiplied element-wise to obtain $I_{\left(0,0\right)}^{ch}$. The final central light field view $I_{\left(0,0\right)}$ is obtained by combining the red, blue, and green color channels.

The combined red, green and blue color channels output the reconstructed light field sub-aperture image at the viewpoint $\left(s^{*},t^{*}\right)$ as $I_{\left(s^{*},t^{*}\right)}$. Figure 6 illustrates the reconstruction of the central light field view for $\left(s^{*},t^{*}\right)=\left(0,0\right)$. All the 169 light field views are similarly reconstructed. The pseudo code of the proposed scheme is given in Algorithm 1. The main steps of BK-SVD decomposition are summarized in Algorithm 2.

**Algorithm 1:**Proposed scheme

**Input**: Light Field L(s,t,u,v)


**Output**: Reconstructed SAIs $I_{\left(s^{*},t^{*}\right)}$, $s^{*},t^{*}\in Z$, $-6\leq s^{*},t^{*}\leq 6$

**1**

Extract SAIs from Light Field: L(s,t,u,v)→ SAIs

**2**

Transform SAIs to multiplicative layers using CNN: SAIs $\to M_{-1},M_{0},M_{1}$

**3**


$M_{-1},M_{0},M_{1}\to B^{ch},ch\in \left\{r,g,b\right\}$


**4**

Find low-rank approximation: Algorithm 2 ($\mathrm{Input}:B^{ch}$, $\mathrm{Output}:W^{ch}$)

**5**

Rearrange and obtain approximated layers: $W^{ch}\to {\hat{M}}_{z}$

**6**

HEVC encode the approximated layers ${\hat{M}}_{z}$

**7**

HEVC decode ${\hat{M}}_{z}\to {\overset{‵}{M}}_{z}$

**8**

Translate for every view $\left(s^{*},t^{*}\right)$: ${\overset{‵}{M}}_{z}\left(u+zs^{*},v+zt^{*}\right)\to {\overset{‶}{M}}_{z(s^{*},t^{*})}\left(u,v\right)$

**9**

Obtain color channel of SAI: ${\overset{‶}{M}}_{-1(s^{*},t^{*})}^{ch}⊙{\overset{‶}{M}}_{0(s^{*},t^{*})}^{ch}⊙{\overset{‶}{M}}_{1(s^{*},t^{*})}^{ch}\to I_{\left(s^{*},t^{*}\right)}^{ch}$

**10**

Combine the color channels to reconstruct the SAIs: $I_{\left(s^{*},t^{*}\right)}$


**Algorithm 2:**Block Krylov Singular Value Decomposition

**Input**: $B\in R^{c\times d}$, error ϵ∈(0,1), rank k≤c,d


**Output**: $W\in R^{c\times k}$

**1**

$q:=\tilde{O}\left(\frac{\log d}{\sqrt{\epsilon }}\right)$, $\Pi ∼N{\left(0,1\right)}^{d\times k}$

**2**


$K:=\left[B\Pi \;\;,\;\;\left(BB^{T}\right)B\Pi \;\;,\;\cdot \cdot \cdot \;,\;\;{\left(BB^{T}\right)}^{q}B\Pi \right]$


**3**

Orthonormalize the columns of K to obtain $Q\in R^{c\times qk}$

**4**

Compute $S:=Q^{T}BB^{T}Q\in R^{qk\times qk}$

**5**

Set ${\bar{U}}_{k}$ to the top *k* singular vectors of S.

**6**


**return** $W=Q{\bar{U}}_{k}$



## 3. Results and Analysis

**Figure 7:**
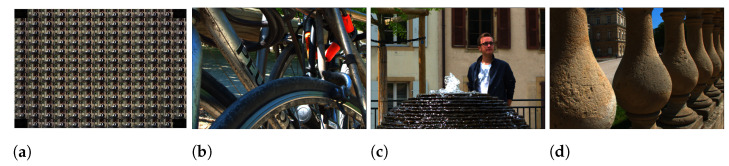
(**a**) The extracted 13 × 13 views of the *Fountain-Vincent2*; central view of (**b**) *Bikes*; (**c**) *Fountain-Vincent2*; (**d**) *Stone-Pillars Outside*.

The performance of the proposed compression scheme was evaluated on real light fields captured by plenoptic cameras. We experimented with *Bikes*, *Fountain-Vincent2*, and *Stone-Pillars Outside* light field images from the EPFL Lightfield JPEG Pleno database [56]. The raw plenoptic images were extracted into 15 × 15 sub-aperture images (each with a resolution of 434 × 625 pixels) using MATLAB Light field toolbox [57]. In Liu et al. [23] and Ahmad et al. [25], only the central 13 × 13 views of the light field were considered as the pseudo sequence for compression. The border SAIs suffer from severe geometric distortion (due to light field lenslet structure) and blurring, and they are less useful in recovering the light field. To facilitate a fair comparison with these state-of-the-art coding methods, we have also discarded the border SAIs and only considered the inner 13 × 13 light field views for our tests. Figure 7 shows the extracted views and the central views of the chosen light field images.

### 3.1. Experimental Settings of CNN

In the first component of the proposed coding scheme (*BLOCK I* in Figure 2), a convolutional neural network learns the optimized multiplicative layers from real light field data. We experimented on the 5 × 5 *Bunnies* dataset [49] to analyze the CNN learning for various hyperparameters. The model was trained with all the following combinations:

- Learning rate (LR): 0.001, 0.0001, 0.00001;
- Batch size (BS): 15, 30;
- Number of epochs (E): 5, 10, 15, 20.

Apart from these combinations of hyperparameters, we analyzed the influence of the network on results by varying the number of convolutional layers as 15, 20, and 25. Maruyama et al. [14] had demonstrated 3 × 3 as an optimal filter size in the network and used 64 channels for the intermediate feature maps. We also chose the same filter size and number of channels in the intermediate layers.

**Figure 8:**
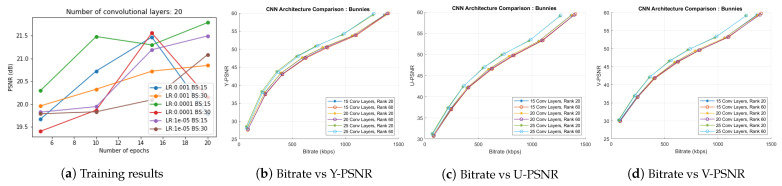
(**a**) Training results of the CNN with 20 convolutional layers. Optimal PSNR is observed for model run for 20 epochs with learning rate 0.0001 and batch size 15; (**b**–**d**) rate-distortion curves comparing results of CNN structures with 15, 20, and 25 convolutional layers trained for 20 epochs with learning rate 0.0001 and batch size 15.

Figure 8a presents the training results of the experiment with 20 convolutional layers in the CNN. We examined similar hyperparameter variation plots for 15 and 25 convolutional layers and concluded that LR 0.0001, BS 15, and E 20 give optimal PSNR results in *BLOCK I* of the proposed scheme. The multiplicative layers produced by the 15, 20, and 25 convolutional layered networks with these choices of hyperparameters were further passed to *BLOCK II* of the proposed coding scheme. We performed the BK-SVD (for ranks 20 and 60) and HEVC step (with QPs 2, 6, 10, 14, 20, 26, 38). Figure 8b–d display the corresponding bitrate versus PSNR results. Thus, we inferred a suitable model for the proposed scheme by choosing 20 convolutional layers and trained the CNN for 20 epochs at a learning rate of 0.0001, and a batch size of 15. Multiplicative layers produced using this model for *Bunnies* are shown in Figure 3.

The multiplicative layers can further be fed into BLOCK II (BK-SVD step + HEVC) for compression. The proposed coding scheme achieves flexible bitrates for the various ranks of BK-SVD of multiplicative layers and varying HEVC quantization parameters. Thus, it realizes the goal of covering a range of multiple bitrates using a single CNN model.

### 3.2. Implementation Details of Proposed Scheme

We implemented the proposed coding scheme on a single high-end HP OMEN X 15-DG0018TX Gaming laptop with 9th Gen i7-9750H, 16 GB RAM, RTX 2080 8 GB Graphics, and Windows 10 operating system. The multiplicative layer patterns for input light fields *Bikes*, *Fountain-Vincent2*, and *Stone-Pillars Outside* were optimized using the chosen CNN with 20 2-D convolutional layers stacked in a sequence. The network was trained (learning rate 0.0001, batch size 25, 20 epochs) with 30 training images generated from light fields *Friends 1*, *Poppies*, *University*, *Desktop*, and *Flowers*, from the EPFL Lightfield JPEG Pleno database [56]. Each training sample was a set of 169 2-D image blocks with 64 × 64 pixels extracted from the same spatial positions in a light-field dataset. We implemented the entire network using the Python-based framework, Chainer (version 7.7.0).

**Figure 9:**
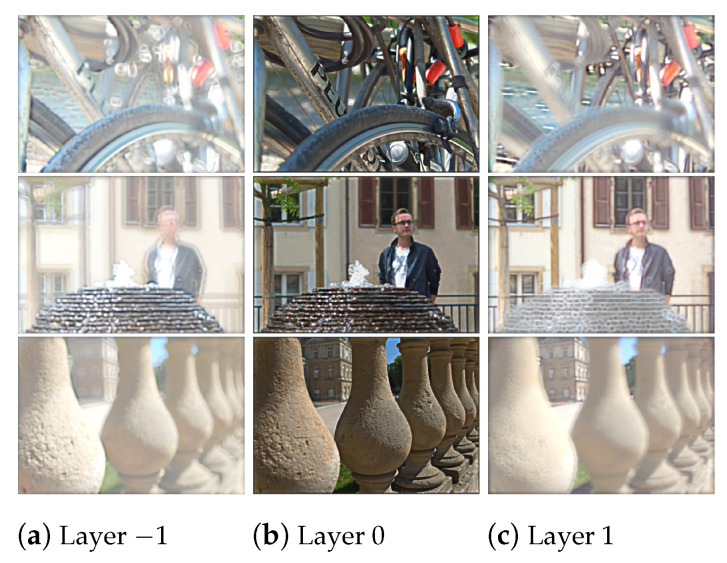
The three multiplicative layers of *Bikes*, *Fountain-Vincent2*, and *Stone-Pillars Outside* light fields obtained from the convolutional neural network.

During training, the CNN (in *BLOCK I* of Figure 2) solves the optimization problem (Equation (5)) by computing loss as the mean square difference between the original light field and the light field reconstructed from multiplicative layers. The optimal multiplicative layers are learned as a result of the optimization of the loss function and this eliminates the need for any ground truth information. The resultant output layers for the *Bikes*, *Fountain-Vincent2*, and *Stone-Pillars Outside* light field images, obtained from the trained CNN model are presented in Figure 9. We rearranged the color channels of these multiplicative layers as described in Section 2.2. We then applied BK-SVD for ranks 4 to 60 (incremented in steps of four) and computed 50 iterations for each rank. The approximated matrices were then rearranged back into layers and converted into the YUV420 color space.

**Figure 10:**
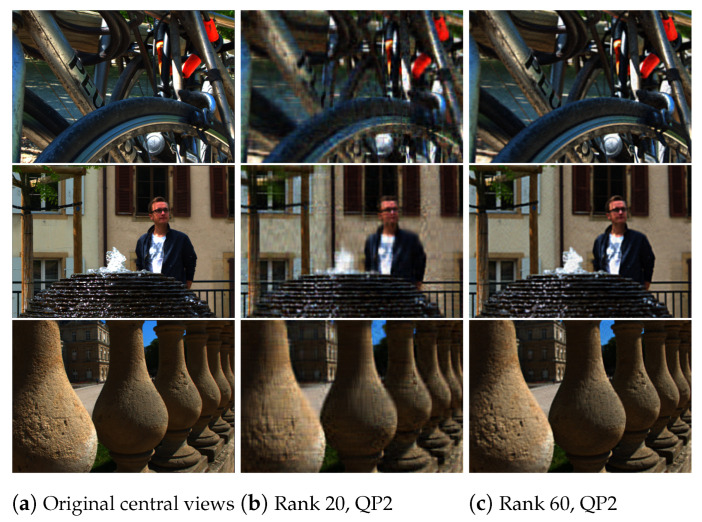
Comparison of the original and reconstructed views. (**a**) Original central views of the three datasets; (**b**) reconstructed central views using our proposed scheme for rank 20 and QP 2; (**c**) reconstructed central views using our proposed scheme for rank 60 and QP 2.

For the HEVC step, we used 32 bit HM encoder (version 11.0) to compress all the YUV files of different approximated ranks. We choose seven quantization parameters, QP 2, 6, 10, 14, 20, 26, and 38, to test both high and low bitrate cases. The encoder compression produces a bitstream for a fixed rank and QP, that can be stored or transmitted. We followed the reverse procedure on the compressed bitstream to reconstruct the 13 × 13 views of the light fields. The HM decoder is used to recover the files in the YUV420 format, which are then converted back to the RGB color space. The three decoded layers are used to reconstruct the entire sub-aperture images of the light field. A comparison of the original central view of the *Bikes*, *Fountain-Vincent2*, and *Stone-Pillars Outside* light field images and the reconstructed central light field views is shown in Figure 10.

### 3.3. Results and Comparative Analysis

**Figure 11:**
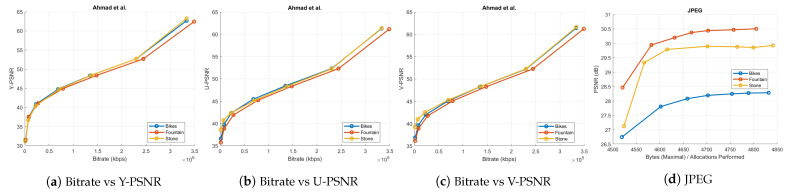
(**a**–**c**) The bitrate versus PSNR graphs of Ahmad et al. coding scheme for all three datasets; (**d**) graph depicting the maximal bytes per allocations performed against the PSNR for *Bikes*, *Fountain-Vincent2*, and *Stone-Pillars Outside* datasets using the JPEG codec.

**Figure 12:**
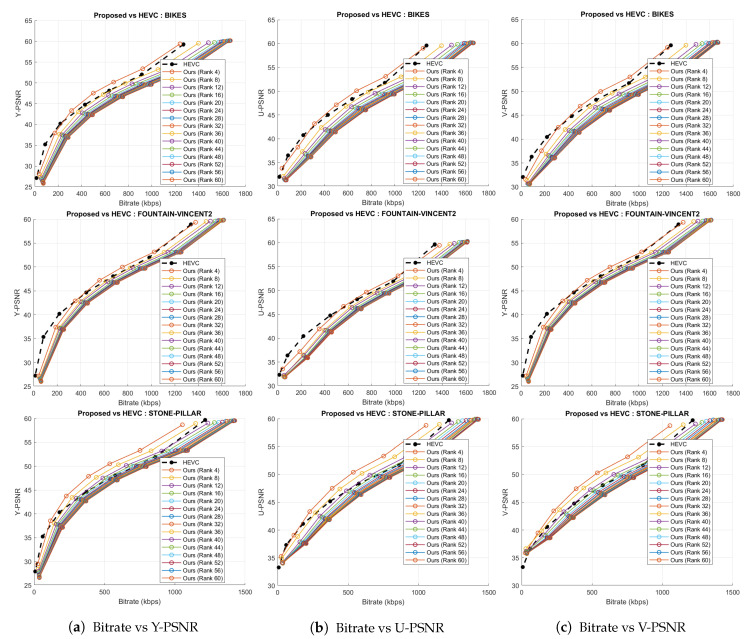
Rate-distortion curves for the proposed compression scheme and HEVC codec for the three datasets.

The performance of our proposed coding scheme is compared to previous pseudo-sequence-based compression schemes [23,25], the HEVC software (version 11.0) [29], and the JPEG standard [58]. We subjected all these anchor schemes to the same test conditions and quantization parameters (QPs-2, 6, 10, 14, 20, and 38) as used in our experimental settings. All PSNR-bitrate graphs are shown in Figure 11 and Figure 12. The total number of bytes written to file during compression by these four anchors is documented in Table 1. Total number of bytes used by the proposed coding scheme for various ranks is summarized in Table 2.

Ahmad et al. [25] interpreted the sub-aperture views of the light field as frames in a multi-view sequence. They availed the tools in the multi-view extension of HEVC to exploit the 2D inter-view correlation among the views. We used the PPM color image format of the inner 13 × 13 views of the *Bikes*, *Fountain-Vincent2*, and *Stone-Pillars Outside* light field images to evaluate this multi-view compression algorithm. The bitrate versus PSNR results of Ahmad et al. is shown in Figure 11a–c. We coded the input SAIs of the chosen light field data with the legacy JPEG [58], as well. The corresponding bytes per allocation performed versus PSNR curve is in Figure 11d.

Liu et al. [23] formulated a predictive coding approach and treated light field views as a pseudo-sequence-like video. They compressed the central view first and then the remaining views in a symmetric, 2D hierarchical order. Motion estimation and compensation in video coding systems were reused to perform inter-view prediction. Due to the public unavailability of Liu et al.’s lenslet YUV files for datasets apart from *Bikes*, we analyzed their compression performance [23] on the 13 × 13 SAIs of *Bikes* only. The total number of bytes used in compression using this approach is given in Table 1.

To evaluate the HEVC [29] performance, the 169 SAIs in YUV420 color space were directly fed into the 32 bit HM codec (version 11.0) for the same chosen QPs. The rate-distortion graphs comparing HEVC with the proposed scheme is shown in Figure 12. Bitrates in case of Ahmad et al. [25] are in the order of $10^{5}$. Hence, these results are plotted separately in Figure 11a–c because of the scale mismatch with bitrates of our proposed scheme. In addition, in case of JPEG, we have shown the maximal bytes per allocations performed versus PSNR plot (Figure 11d) and the x-axis is not the same as in Figure 12. Thus, for a fair comparison, we have not plotted the results of the proposed coding scheme and JPEG together. The proposed coding scheme consistently outperforms on all datasets.

**Figure 13:**
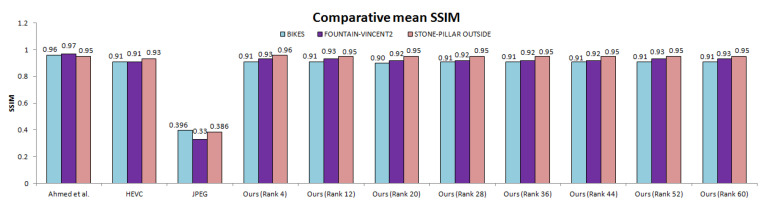
Comparative mean SSIM for the proposed scheme and anchor coding methods. Average SSIM was computed over all 169 views and all quantization parameters.

The total number of bytes taken by our approach is comparatively much less than other anchors (Table 1 and Table 2) for both lower and higher ranks. We also computed SSIM (Structural Similarity Index) scores for all 13 × 13 views of scenes. Figure 13 illustrates the comparative mean SSIM values of the proposed coding scheme and results of Ahmad et al. [25], HEVC [29], and JPEG [58] codecs. We saved significant bitrates at different ranks and maintain good reconstruction quality.

Further, we performed an objective assessment using the Bjontegaard [59] metric. This metric can compare the performance of two different coding techniques. We compared bitrate reduction (BD-rate) of proposed scheme with respect to Ahmed et al., Liu et al., and HEVC codec. The average percent difference in rate change is estimated over a range of quantization parameters for the fifteen chosen ranks.

A comparison of the percentage of bitrate savings of our proposed coding scheme with respect to the anchor methods for the *Bikes*, *Fountain-Vincent2*, and the *Stone-Pillars Outside* datasets are shown in Table 3, Table 4 and Table 5, respectively. On *Bikes* data, the proposed scheme achieves 98.94%, 40.42%, and 81.37% bitrate reduction compared to Ahmad et al., HEVC, and Liu et al., respectively (Table 3). On *Fountain-Vincent2* data, we noticed 99.03% and 35.80% bitrate savings compared to Ahmad et al. and HEVC codec, respectively (Table 4). On *Stone-Pillars Outside* data, we achieve 99.20% and 22.43% bitrate reduction compared to Ahmad et al. and HEVC, respectively (Table 5).

### 3.4. Advantages of Using Multiplicative Layers

The proposed scheme generates three optimal multiplicative layers of input light field for layered light field displays. There is a clear advantage to approximate and encode just the three multiplicative layers rather than the entire set of light field views in terms of computation speed, bytes written to file during compression, bitrate savings with respect to state-of-the-art coding methods, as well as SSIM performance. To analyze this, we experimented by directly feeding all 13 × 13 views of the *Bikes* light field into *BLOCK II* of the proposed coding scheme *(AV(169))*. These results were then compared with usage of just 3 multiplicative layers described in the previous sections *(ML(3))*. Block-Krylov low rank approximation was done for ranks 20 and 60, followed by HEVC encoding for quantization parameters 2, 6, 10, 14, 20, and 38.

We noted the computation time for each step in *BLOCK II*. Table 6 highlights the results. Evidently, the proposed compression scheme works much faster with just three light field multiplicative layers. This proves to be advantageous over using all views of the light field. We have also compared the number of bytes written to file during compression in Table 7, where a higher number of bytes in case of *AV(169)* can be observed. The bitrate reduction of *ML(3)* and *AV(169)* with respect to Liu et al. [23], HEVC codec [29], and Ahmed et al. [25] (estimated over a range of quantization parameters 2, 6, 10, 14, 20, 26, 38 for ranks 20 and 60) is depicted in Table 8.

**Figure 14:**
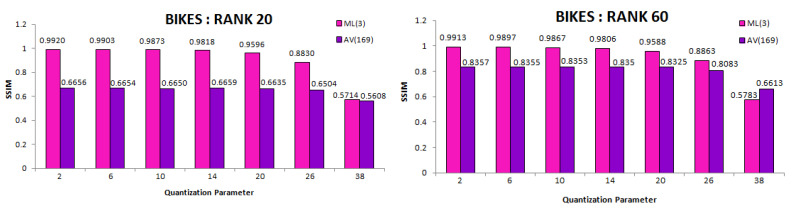
Mean SSIM scores over each QP of decoded views in *BLOCK III* of proposed scheme with *(ML(3))* and without *(AV(169))* multiplicative layers. Experiment evaluated on *Bikes* light field for BK-SVD ranks 20 and 60.

To evaluate the decoding module of the proposed scheme *(BLOCK III)*, we used the SSIM metric to compare decoded views of experiment *AV(169)* and *ML(3)*. Mean SSIM over the decoded views was calculated for each QP result and each rank of *Bikes*. These SSIM comparison graphs are illustrated in Figure 14. The proposed scheme with three multiplicative layers performs better perceptually, as well, since the matrix constructed to perform BK-SVD in case of *AV(169)* is larger in size. The approximation quality deteriorates when all 13 × 13 views are simultaneously considered.

### 3.5. Advantages of Using CNN over Analytical Methods in BLOCK I

**Figure 15:**
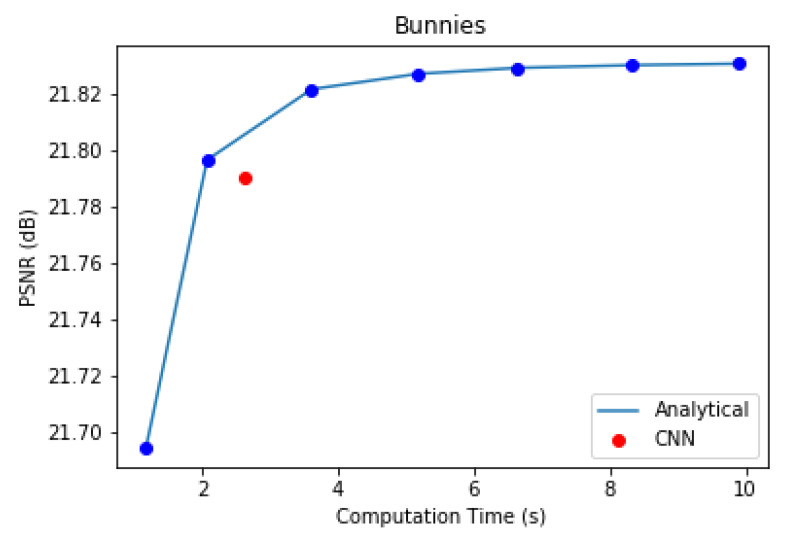
Computation time and accuracy of reproduced light fields using analytical and CNN-based optimization of multiplicative layers.

In the first component of the proposed coding scheme, the multiplicative layered representation of the light field is generated. This can be done analytically one layer at a time, with repetition until the solution of Equation (2) converges, or by using a CNN [14]. We experimented with the 5 × 5 *Bunnies* dataset [49] to compare the performance of the CNN and analytical optimization method to obtain three optimal multiplicative layers. The analytical method was evaluated for 10, 25, 50, 75, 100, 125, and 150 iterations. The CNN used was trained with 20 2-D convolutional layers for 20 epochs at a learning rate of 0.0001 and a batch size of 15. Figure 15 illustrates the PSNR versus computation time plot of this experiment.

In case of the analytical approach, accuracy gradually increases with computation time. The inference time for the CNN in Figure 15 is marginally slower than that of the analytical method for the same PSNR performance in our experiment. Nevertheless, Maruyama et al. [14] have demonstrated that, with better GPUs and more training data, CNN inference can be performed much faster than the analytical method for the same reconstruction quality.

**Figure 16:**
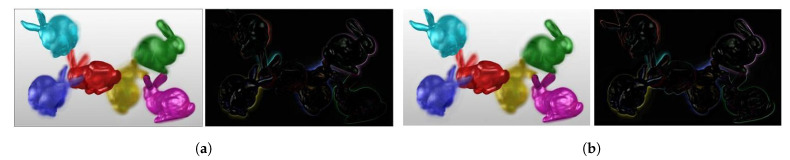
View 19 of *Bunnies* reproduced using analytical (ANA) and CNN-based (CNN) optimization of multiplicative layers, with corresponding difference images. (**a**) ANA: Reproduced view and error, PSNR: 19.94 dB, SSIM: 0.895; (**b**) CNN: Reproduced view and error, PSNR: 22.18 dB, SSIM: 0.918.

We have also shown view 19 of the *BLOCK I* reproduced *Bunnies* light field using analytical and CNN methods, along with their error images, in Figure 16. The CNN results are better in this case in terms of PSNR and SSIM scores, as well. Thus, we prefer the CNN over the analytical method in *BLOCK I* of the proposed coding scheme as it strikes a better balance between computation speed and accuracy.

## 4. Conclusions

We proposed an efficient representation and novel lossy compression scheme for layered light-field displays with light-ray operations regulated using multiplicative layers and a CNN-based method. A single CNN model is employed to learn the optimized multiplicative layer patterns. A light field is compactly (compressed) represented on a Krylov subspace, considering the transmittance patterns of such a few layers and approximation derived by Block Krylov SVD. Factorization derived from BK-SVD exploits spatial correlation in multiplicative layer patterns with varying low ranks. Further, encoding with HEVC eliminates the inter-layer and intra-layer redundancies in the low-rank approximated representation. By choosing varying ranks and quantization parameters, the scheme is flexible to adjust the bit rates in the layered representation with a single CNN. Consequently, it realizes the goal of covering a range of multiple bitrates within a single trained CNN model. The experiments with benchmark light field datasets exhibit very competitive results.

This critical characteristic of the proposed scheme sets it apart from other existing light field coding methods which train a system (or network) to support only specific bitrates during the compression. Moreover, current solutions are not specifically designed to target layered displays. Broadly, compression approaches are classified to work for lenslet-based formats or sub-aperture images based pseudo-sequence representation. Our proposed scheme could flexibly work with different light-ray operations (multiplication and addition) and analytical or data-driven CNN-based methods, targeting multi-layered displays. It is adaptable for a variety of multi-view/light field displays. In addition, it can complement existing light-field coding schemes, which employ different networks to encode light field images at different bit rates. This would enable deploying the concept of layered displays on different auto-stereoscopic platforms. One can optimize the bandwidth for a given target bit-rate and deliver the 3D contents with limited hardware resources to best meet the viewers’ preferences.

Our future work span in several directions. We will further extend the proposed idea to light field displays, such as ones with more than three light attenuating layers, projection-based, and holographic displays with optical elements constructed using additive layers. Proof-of-concept experiments with our scheme pave way to form a deeper understanding in the rank-analysis of a light field using other mathematically valid tensor-based models and efficient layered representations for 3D displays. Another interesting direction is to extend the proposed mathematical formulation for coded mask cameras useful in compression of dynamic light field contents or a focal stack instead of processing multi-view images in layered displays. We aim to verify our scheme with not only computer simulations, but with a physical light field display hardware. In addition, we will consider the aspect of human perception by performing a subjective analysis of perceptual quality on a light field display. The proposed algorithm uses RGB space in which all channels are equally important. For image perception, there are better color spaces, like HSV/HSL or Lab. Analyzing other color spaces would be worth considering in further work.

## Figures and Tables

**Table 1:** The total number of bytes written to file during compression for three datasets using JPEG, Ahmad et al., Liu et al., and HEVC codec.

SCENE	QP	JPEG		Ahmad et al.	HEVC	Liu et al.
		Bytes Maximal	Allocations Performed			
Bikes	2	1,289,500,060	285,334	24,312,508	26,855,413	13,236,265
	6	1,282,545,982	278,698	16,769,702	19,348,737	8,502,825
	10	1,277,959,454	274,320	9,845,153	13,443,535	4,325,040
	14	1,274,522,080	271,040	5,017,757	9,081,969	1,806,765
	20	1,270,588,768	267,288	1,702,024	4,612,312	531,260
	26	1,267,960,215	264,780	578,837	1,872,858	186,890
	38	1,264,811,424	261,776	109,882	309,894	49,819
Fountain-Vincent2	2	1,289,376,407	285,222	25,458,464	28,247,570	-
	6	1,284,189,141	280,268	17,822,580	20,753,794	-
	10	1,280,142,984	276,406	10,784,296	14,296,728	-
	14	1,277,256,515	273,654	5,734,607	9,448,082	-
	20	1,274,473,022	270,998	2,005,209	4,611,589	-
	26	1,270,323,433	267,038	608,622	1,778,323	-
	38	1,266,732,371	263,612	107,314	282,926	-
Stone-Pillars Outside	2	1,289,107,839	284,980	24,318,932	25,687,041	-
	6	1,285,439,994	281,480	16,763,791	18,184,046	-
	10	1,281,396,834	277,622	9,966,816	12,175,243	-
	14	1,274,580,935	271,118	5,129,148	7,881,904	-
	20	1,269,731,759	266,490	1,613,407	3,795,400	-
	26	1,267,162,562	264,038	499,315	1,266,845	-
	38	1,264,130,704	261,144	81,220	165,209	-

**Table 2:** The total number of bytes written to file during compression using our proposed scheme for fifteen chosen ranks.

SCENE	QP	Rank 4	Rank 8	Rank 12	Rank 16	Rank 20	Rank 24	Rank 28	Rank 32	Rank 36	Rank 40	Rank 44	Rank 48	Rank 52	Rank 56	Rank 60
Bikes	2	465,324	524,323	556,265	575,476	589,749	596,927	603,836	609,989	612,874	617,204	619,711	621,985	624,307	625,418	626,699
	6	345,970	394,844	422,644	440,498	454,163	462,476	468,236	473,869	479,041	482,762	485,010	487,279	490,158	491,914	493,319
	10	252,714	289,578	311,384	325,476	335,791	342,412	348,215	353,720	358,598	362,391	365,720	367,504	370,269	371,874	373,835
	14	187,722	219,282	236,455	247,443	254,629	259,664	264,641	268,640	270,989	274,188	276,139	277,926	279,651	280,930	282,329
	20	117,907	139,701	152,559	160,308	165,537	170,361	173,749	176,666	178,642	179,836	181,396	182,388	184,061	185,106	185,288
	26	64,705	79,211	87,522	92,133	96,011	98,170	99,659	101,487	102,731	103,588	104,649	104,932	106,117	106,529	107,238
	38	13,892	18,321	20,884	22,785	23,855	24,670	25,376	26,045	26,203	26,600	27,171	27,196	27,673	27,546	27,562
Fountain-Vincent2	2	516,160	549,485	563,480	578,844	586,229	590,485	595,204	597,747	598,965	602,633	602,416	603,626	604,461	605,605	605,053
	6	384,173	415,523	428,058	442,982	449,339	454,349	458,496	461,966	464,439	465,384	467,293	467,920	468,986	469,543	470,570
	10	282,959	308,182	317,127	329,436	335,826	340,236	343,505	347,469	349,302	350,126	352,036	352,735	354,723	354,932	355,663
	14	210,399	231,072	237,650	247,782	252,603	255,323	257,685	260,650	262,153	262,876	264,412	264,870	265,828	266,672	267,453
	20	132,576	148,200	153,252	160,184	163,285	165,605	167,499	169,054	169,138	170,198	170,229	170,302	171,155	172,099	172,095
	26	70,632	80,893	83,555	88,718	90,406	91,399	92,972	93,937	94,522	94,618	95,285	95,368	95,402	95,757	96,129
	38	15,944	18,976	20,430	21,418	21,960	22,430	22,792	23,024	23,241	23,419	23,545	23,529	23,815	23,721	23,750
Stone-Pillars Outside	2	395,160	430,554	463,224	480,429	492,073	501,838	510,784	515,002	519,969	523,496	526,519	529,851	531,759	533,523	534,984
	6	282,249	311,619	340,148	356,242	367,825	377,322	383,633	389,337	394,027	398,591	400,673	403,800	405,328	407,812	409,376
	10	201,642	224,608	245,521	257,421	265,412	272,566	278,653	282,513	286,930	289,816	292,090	295,110	296,522	298,174	299,145
	14	145,152	165,716	183,136	192,439	198,885	204,685	207,946	211,555	213,927	215,770	217,476	2189,64	219,533	220,136	222,381
	20	85,558	100,871	112,482	118,850	122,415	125,708	128,209	130,955	132,507	134,048	134,861	136,701	136,890	137,469	138,307
	26	43,278	53,407	59,221	63,628	66,177	68,108	69,342	70,701	72,331	72,935	73,700	74,077	74,670	75,747	76,062
	38	9478	10,984	11,799	12,521	12,899	13,163	13,472	13,589	13,655	13,632	13,732	13,801	13,933	13,956	13,870

**Table 3:** Bjontegaard percentage rate savings for the proposed compression scheme with respect to Ahmad et al., HEVC codec, and Liu et al. (negative values represent gains) on *Bikes* data.

	Ahmad et al.			HEVC			Liu et al.		
Rank	Y	U	V	Y	U	V	Y	U	V
4	−99.251481	−99.276458	−99.274087	−5.881261	0.180365	−11.739816	−67.966775	−71.264162	−70.635424
8	−99.090493	−99.121327	−99.143757	−25.484779	−25.959331	−27.984525	−74.782197	−78.380012	−76.773975
12	−98.999026	−99.034508	−99.068975	−33.773506	−34.641588	−35.470272	−77.639238	−80.996109	−79.166755
16	−98.939151	−98.986041	−99.026115	−38.00838	−38.778121	−39.062217	−79.199071	−82.263679	−80.394787
20	−98.899136	−98.948792	−98.996347	−40.60945	−41.390099	−41.342234	−80.145098	−83.146997	−81.1756
24	−98.871015	−98.930821	−98.967791	−42.383595	−42.746015	−42.750932	−80.793824	−83.601011	−81.803752
28	−98.853191	−98.911292	−98.951353	−43.639337	−43.901611	−44.03624	−81.262957	−83.968503	−82.238102
32	−98.832965	−98.890869	−98.93992	−44.748237	−44.949141	−45.081394	−81.66317	−84.296064	−82.651585
36	−98.819182	−98.884722	−98.921536	−45.532868	−45.35228	−45.574059	−81.967138	−84.506536	−82.849684
40	−98.807443	−98.875328	−98.918501	−46.250925	−46.02426	−46.029313	−82.231804	−84.731675	−82.987648
44	−98.797027	−98.863221	−98.906404	−46.792409	−46.647476	−46.641298	−82.426399	−84.908654	−83.197302
48	−98.792277	−98.857093	−98.8995	−47.142026	−46.930989	−46.844444	−82.556527	−85.038781	−83.297143
52	−98.780806	−98.847028	−98.899573	−47.652849	−47.562722	−47.213633	−82.750674	−85.198862	−83.459447
56	−98.77693	−98.838412	−98.894099	−47.880471	−47.719856	−47.340388	−82.832358	−85.296081	−83.518001
60	−98.77114	−98.836726	−98.885478	−48.133692	−47.798751	−47.625073	−82.932904	−85.334731	−83.607177
**Average**	−98.88541753	−98.94017587	−98.9795624	−40.260919	−40.01479167	−40.9823892	−80.0766756	−82.8621238	−81.1837588

**Table 4:** Bjontegaard percentage rate savings for the proposed compression scheme with respect to Ahmad et al. and HEVC codec (negative values represent gains) on *Fountain-Vincent2* data.

	Ahmad et al.			HEVC		
Rank	Y	U	V	Y	U	V
4	−99.183799	−99.229844	−99.264954	−21.521969	−13.921367	−5.773992
8	−99.086069	−99.127875	−99.17076	−31.169436	−30.225204	−21.630714
12	−99.050404	−99.103297	−99.145429	−34.314673	−32.308096	−24.616496
16	−99.005551	−99.063528	−99.100118	−37.4743	−36.182694	−30.309729
20	−98.984593	−99.046354	−99.080496	−39.061992	−37.607852	−32.701631
24	−98.971614	−99.030753	−99.071674	−40.004309	−38.543402	−33.089425
28	−98.957412	−99.020463	−99.059243	−40.851959	−39.456734	−34.472345
32	−98.948131	−99.012691	−99.046828	−41.484072	−39.915501	−35.497824
36	−98.942413	−99.008685	−99.039732	−41.862561	−40.141429	−35.856707
40	−98.937226	−99.004562	−99.039085	−42.18118	−40.524649	−36.136539
44	−98.932066	−98.996273	−99.037422	−42.540685	−41.006276	−35.888463
48	−98.92917	−99.000517	−99.034159	−42.713349	−40.941182	−35.891383
52	−98.926749	−98.990882	−99.03173	−42.974712	−41.265832	−36.465998
56	−98.926221	−98.99509	−99.026921	−43.069367	−41.361922	−36.529031
60	−98.92157	−98.990063	−99.032116	−43.239617	−41.513125	−36.632594
**Average**	−98.9801992	−99.0413918	−99.07871113	−38.96427873	−36.994351	−31.43285807

**Table 5:** Bjontegaard percentage rate savings for the proposed compression scheme with respect to Ahmad et al. and HEVC codec (negative values represent gains) on *Stone-Pillars Outside* data.

	Ahmad et al.			HEVC		
Rank	Y	U	V	Y	U	V
4	−99.456179	−99.465611	−99.464082	12.433281	16.684821	28.649503
8	−99.341767	−99.380922	−99.406866	−11.673143	−2.061917	18.073502
12	−99.262494	−99.290023	−99.322609	−22.699987	−19.492942	1.333068
16	−99.218085	−99.251555	−99.280718	−27.726039	−24.244093	−5.509125
20	−99.18522	−99.227969	−99.248049	−30.848873	−26.809427	−10.780161
24	−99.157964	−99.202939	−99.225599	−33.281782	−29.211062	−13.569113
28	−99.139248	−99.18847	−99.20702	−35.037932	−30.350017	−16.196362
32	−99.120267	−99.171885	−99.193537	−36.513559	−31.837999	−17.08197
36	−99.103079	−99.162032	−99.179835	−37.675867	−33.229377	−19.124177
40	−99.093228	−99.152927	−99.171138	−38.506503	−33.604077	−19.682844
44	−99.083238	−99.151691	−99.165229	−39.251178	−33.54724	−20.452764
48	−99.075932	−99.138257	−99.157717	−39.744394	−34.621605	−20.963765
52	−99.068293	−99.140571	−99.155556	−40.293038	−34.355048	−21.187093
56	−99.065237	−99.131556	−99.152072	−40.583026	−35.087053	−21.289896
60	−99.058314	−99.128089	−99.146575	−40.991487	−35.288201	−22.048587
**Average**	−99.161903	−99.2122998	−99.23177347	−30.82623513	−25.80368247	−10.65531893

**Table 6:** Comparison of computation speed in *BLOCK II* of proposed scheme with *(ML(3))* and without *(AV(169))* multiplicative layers. Experiment evaluated on *Bikes* light field for BK-SVD ranks 20 and 60.

		Computation Time (s)							
Input	Rank	BKSVD	HEVC QP2	HEVC QP6	HEVC QP10	HEVC QP14	HEVC QP20	HEVC QP26	HEVC QP38
ML(3)	20	1.399	38.892	37.087	33.936	31.876	28.261	23.874	15.643
AV(169)	20	37.117	2592.1	3450.8	3806.8	2327.6	1360.4	1010.6	659.835
ML(3)	60	1.711	38.937	37.136	34.644	32.183	28.355	23.811	16.101
AV(169)	60	47.22	3068.9	2552.1	2261.8	1925.15	1460.3	1074.9	680.548

**Table 7:** Comparison of bytes written to file during compression using proposed scheme with *(ML(3))* and without *(AV(169))* multiplicative layers. Experiment evaluated on *Bikes* light field for BK-SVD ranks 20 and 60.

	ML(3)	AV(169)	ML(3)	AV(169)
QP	Rank 20	Rank 20	Rank 60	Rank 60
2	589,749	21,281,614	626,699	26,428,959
6	454,163	14,426,779	493,319	19,099,470
10	335,791	9,712,428	373,835	13,117,452
14	254,629	6,287,936	282,329	8,594,436
20	165,537	3,070,874	185,288	4,197,244
26	96,011	1,235,911	107,238	1,651,635
38	23,855	209,829	27,562	266,666

**Table 8:** Bjontegaard percentage rate savings for the proposed compression scheme with respect to Liu et al., HEVC codec, and Ahmad et al. on *Bikes* data with *(ML(3))* and without *(AV(169))* multiplicative layers for ranks 20 and 60 (negative values represent gain over anchor).

		Liu et al.			HEVC			Ahmad et al.		
Input	Rank	Y	U	V	Y	U	V	Y	U	V
ML(3)	20	−80.145	−83.14	−81.17	−40.6	−41.39	−41.34	−98.89	−98.94	−98.99
AV(169)	20	−36.78	−50.35	−48.36	64.76	56.71	53.86	−99.5	−99.49	−99.49
ML(3)	60	−82.93	−85.33	−83.6	−48.13	−47.79	−47.62	−98.77	−98.83	−98.88
AV(169)	60	−59.46	−69.2	−67.49	11.08	6.145	5.98	−99.28	−99.29	−99.3

## Data Availability

Not applicable.

## Abbreviations

The following abbreviations are used in this manuscript:

LCD	Liquid crystal display
CNN	Convolutional neural network
BK-SVD	Block Krylov singular value decomposition
HEVC	High efficiency video coding
QP	Quantization parameter
HOE	Holographic optical element
HLRA	Homography-based low-rank approximation
GNN	Gaussian neural network
SAI	Sub-aperture image
PSNR	Peak signal-to-noise ratio
SSIM	Structural similarity index
